# Circumcision and Its Abuses

**Published:** 1909-07-03

**Authors:** 


### Diseases of Children.

### CIRCUMCISION AND ITS ABUSES.

It is not sufficiently realised that, however advisable circumcision is on hygienic grounds, the anatomical state of the foreskin is by no means frequently sufficient justification for operating. There is much too great a tendency to regard a long and narrow foreskin as in itself a proof that circumcision is needed. Such a foreskin is the characteristic of male babies at birth; while, on the other hand, the penis of the new-born infant is small in size, and frequently very small.

In the new-born babe the glans and prepuce are adherent by reason of the persistence of the epithelial agglutination of the surfaces. Few babies are born with the adhesions fully separated, but separation takes place in the course of some months, and the perfect adult condition is attained about the eighth year.

True congenital phimosis is a rare condition. Sometimes the orifice is constricted, and occasionally it is absent. Constriction of the orifice may lead to ballooning of the foreskin on micturition, a very evident sign, which quickly attracts the attention of the nurse, for the state of the foreskin in babies seems to be peculiarly interesting to nurses, and the question of circumcision generally arises through their initiative. It is stated that a constricted orifice may lead to dilatation of the urethra, bladder, ureters, and kidneys, giving rise to hydronephrosis and atrophy of the renal tissue. Certainly such results are extremely rare from this cause, but excite undue apprehension in the mind of the family doctor. It is also said to cause the retention, accumulation and decomposition of smegma, eczema and balanitis, preputial calculi, adhesion of the prepuce, narrowing of the meatus, urethritis, cystitis, pyelitis, retention, incontinence, and enuresis.

To the local irritation which accompanies some of these affections are ascribed restlessness, insomnia, irritability, paroxysmal screaming attacks, pavor nocturnus, dysuria, frequent micturition, severe colic, and even pain in the hip. Painful micturition is much more probably due to highly acid urine. Masturbation has followed on local irritation, but, on the other hand, the habit is by no means rare in the circumcised, and has often been ascribed to the effect of circumcision and the friction of clothes on the sensitive glans. Continued mild inflammatory mischief leads to adhesions and the development of a thickened non-retractile foreskin, with subsequent difficulties in coitus and liability to attacks of balanitis. Straining to pass water is supposed to develop or maintain hernia, prolapsus recti, and even hydrocele.

In the course of a very extensive experience of the ailments of infancy the writer has found remarkably little confirmatory evidence of the occurrence of these conditions. Many of them are almost unknown.

Let it be clearly understood that mere redundancy of foreskin is no indication for circumcision. The penis develops later, and subsequently the supposed long foreskin may be insufficient to cover the glans completely. If the prepuce can be retracted with moderate ease, it should certainly be left. It is a very valuable protection for the glans. The fact that among the children of the careless and unwashen smegma may accumulate under the prepuce and become offensive, is not an argument in favour of operation, but a slur on the person responsible for the welfare of the child.

Circumcision must not be regarded as a trivial and harmless operation, for many evil and fatal results have ensued. Sepsis, sloughing of the skin and ultimately extensive scarring, sloughing and gangrene of the penis, fatal hæmorrhage, erysipelas, and pyæmia have all occurred. Syphilis and tuberculosis have been transmitted when the operation has been done as a religious rite, and not by a trained surgeon. Hæmorrhage is rare, for the Jews remove skin only, do not cut the mucous membrane, and carefully avoid the frænum, though neither sutures nor ligatures are used. Hæmorrhage is commonly due to neglect to tie the vessels of the frænum.

Apart, however, from serious and fatal sequels, the operation of circumcision may be a source of discredit to the operator and of subsequent trouble to the child. It is by no means rare to find an excessive amount of skin removed. A chronically thickened preputial stump or a mass of redundant skin may give the organ an unkempt and ragged appearance, which spoils the reputation of the surgeon for years and is a constant source of gossip among the female branches of the family, although the inartistic appearance eventually is lost or forgotten.

In many babies it is quite sufficient to separate the adhesions with a probe, without causing bleeding. Others can be treated by dilatation with dressing or artery forceps, until the foreskin can be easily retracted. It is then cleaned, oiled, and replaced. Retraction and oiling should be done daily for a time. This may be left to the mother or nurse if the foreskin can be replaced easily. Otherwise there is the prospect of being hastily summoned to deal with a paraphimosis. If the surfaces bleed on separation, adhesion is almost certain to recur, for the retraction cannot be carried out daily without pain and will be neglected. Failing cure by these simple measures, recourse must be had to complete circumcision; to incision of the mucous membrane only on each side; to longitudinal dorsal incision of the foreskin; or to other modification of the complete operation, depending on the length of the foreskin, the degree of adhesion and stenosis, and the ideas of the parents and operator in reference to the desirability of this operation. Care should be taken not to remove too much skin, leaving enough to cover the corona, and to enlarge a narrow meatus, if present; for this may quite well be the cause of screaming and straining on micturition.

